# Developmental Expression and Functions of the Small Heat Shock Proteins in *Drosophila*

**DOI:** 10.3390/ijms19113441

**Published:** 2018-11-02

**Authors:** Teresa Jagla, Magda Dubińska-Magiera, Preethi Poovathumkadavil, Małgorzata Daczewska, Krzysztof Jagla

**Affiliations:** 1 GReD, INSERM U1103, CNRS UMR6293, University of Clermont Auvergne, 28, Place Henri Dunant, 63000 Clermont-Ferrand, France; preethi.poovathumkadavil@uca.fr (P.P.); christophe.jagla@uca.fr (K.J.); 2 Department of Animal Developmental Biology, Institute of Experimental Biology, University of Wroclaw, Wroclaw, Sienkiewicza 21, 50-335 Wroclaw, Poland; magda.dubinska-magiera@uwr.edu.pl (M.D.-M.); malgorzata.daczewska@uwr.edu.pl (M.D.)

**Keywords:** sHsp, *Drosophila*, embryonic development, muscle, nervous system, reproductive system

## Abstract

Heat shock proteins (Hsps) form a large family of evolutionarily conserved molecular chaperones that help balance protein folding and protect cells from various stress conditions. However, there is growing evidence that Hsps may also play an active role in developmental processes. Here, we take the example of developmental expression and function of one class of Hsps characterized by low molecular weight, the small Hsps (sHsps). We discuss recent reports and genome-wide datasets that support vital sHsps functions in the developing nervous system, reproductive system, and muscles. This tissue- and time-specific sHsp expression is developmentally regulated, so that the enhancer sequence of an sHsp gene expressed in developing muscle, in addition to stress-inducible elements, also carries binding sites for myogenic regulatory factors. One possible reason for sHsp genes to switch on during development and in non-stress conditions is to protect vital developing organs from environmental insults.

## 1. Introduction

Heat shock proteins (Hsps) recognize and form complexes with incorrectly folded or denatured proteins, enabling either recovery of correct folding or compartmentalization and degradation of misfolded proteins. As a part of the intrinsic protein quality control machinery, they are synthesized transiently after exposure to heat or other stress conditions. In general, they increase their activity in aged cells, and promote longevity [1]. There is also growing evidence for stress-independent and tissue-specific expression of several members of the Hsp gene family. Recent work in *Drosophila* [2] revealed that several Hsp genes could have vital developmental functions. Among 95 Hsp genes tested, the ubiquitous gene knockdown of 42 of them resulted in F1 lethality, indicating their essential role in fly development.

Small Hsps (sHsps) are among the heat shock proteins most upregulated following stress. They play a crucial role in the maintenance of protein homeostasis, preventing nonspecific aggregation of the substrate protein in an ATP-independent manner [3]. We will focus on sHsps identified in *Drosophila*, in which high-throughput developmental gene expression (www.flyatlas.org) and several functional studies have already been performed [2,4,5,6,7,8]. Importantly, ubiquitous RNAi knockdown of six *Drosophila* sHsps (*Hsp23*, *Hsp26*, *Hsp27*, *CG4461*, *l(2)efl*, and *CG14207*) resulted in lethality [2], suggesting they play vital developmental roles.

## 2. Structural Features of *Drosophila* sHsps

**Figure 1:**
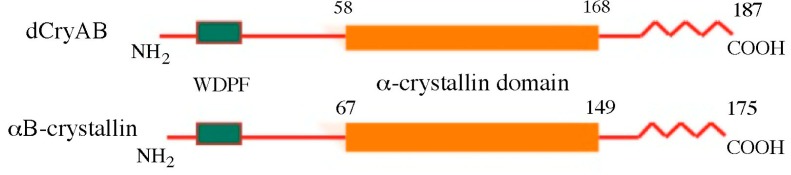
dCryAB as an example of heat shock protein (Hsp) protein domain organization conserved with human αB-crystallin. WDPF and α-crystallin domains are indicated. Numbers refer to amino acid positions.

Classical cloning and genome sequencing have identified 12 sHsp genes in *Drosophila* [3]. Among them, eight (*Hsp22*, *Hsp23*, *Hsp26*, and *Hsp27*, *Hsp67Ba*, *Hsp67Bb*, *Hsp67Bc*, and *CG4461*) are clustered within a 12 kb section of the 67B region on the left arm of the third chromosome [9], and four others (*l(2)efl/dCryAB*, *CG14207*, *CG13133*, and *CG7409*) are all associated with distinct chromosomal regions. Except for *l(2)efl*, *Hsp67Bb*, and *CG14207*, all the other *Drosophila* sHsp genes consist of a simple coding sequence devoid of introns. sHsps encode three major domains of homology, including the 80-amino acid domain, homologous to a mammalian α-crystallin domain [10], located in the carboxy-terminal section of all sHsps. Finally, in all sHsps, with the exception of *Hsp22*, a hydrophobic WDPF domain, potentially involved in client protein binding, is found in the amino-terminal region (see dCryAB protein domain organization as an example—Figure 1). The COOH terminal parts of *Drosophila* Hsps downstream of the α-crystallin domain show differential lengths and amino acid sequence variability with Hsp 23, 26, and 27 sharing a 12-amino acid motif [11]. Regarding evolutionary conservation, except dCryAB/HSPB5 and Hsp67Bc/HSPB8, for the majority of *Drosophila* sHsps, human orthologs have not been clearly defined [10].

## 3. Developmental and Tissue-Specific Regulation of sHsps in *Drosophila*

**Figure 2:**
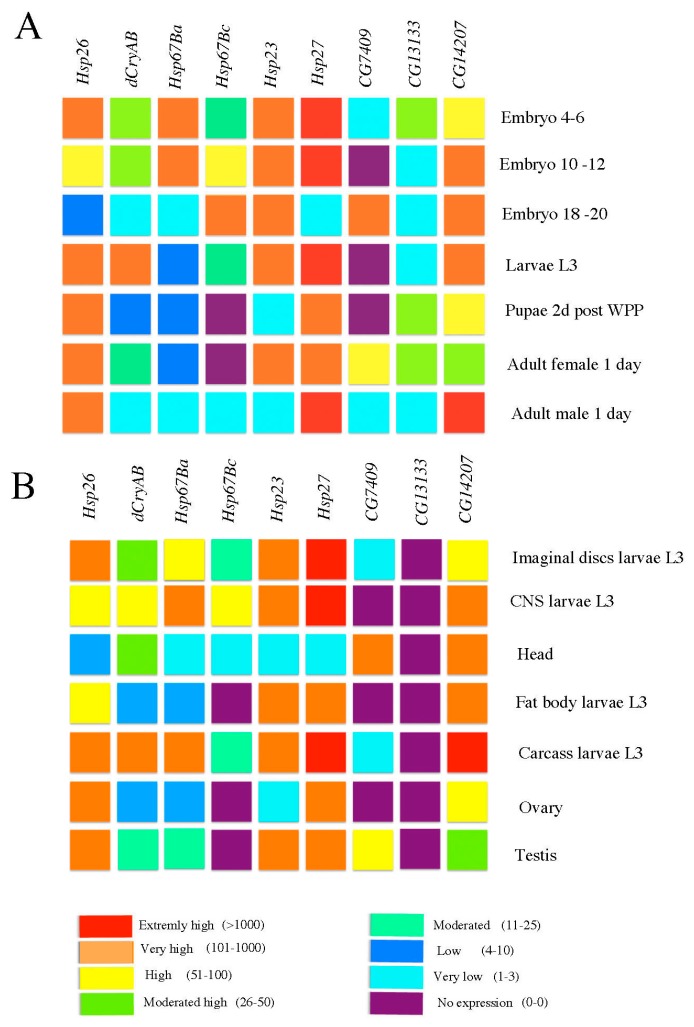
Time (**A**) and tissue-specific (**B**) *Drosophila* sHsp genes expression during development (adapted from the Flyatlas transcriptomic datasets presented in the Flybase). Color code of expression with signal level between brackets is indicated below the figure.

High-throughput transcriptomic studies (www.flyatlas.org; www.flybase.org) indicate that, in addition to coordinately induced expression in response to a heat stress, almost all the *Drosophila* sHsps show a specific spatial and/or temporal pattern of expression during development (Figure 2).

For example, *Hsp23*, *Hsp26*, *Hsp27*, and *CG7409* show high to very high transcript levels in the testis, whereas *CG14207*, *Hsp26*, and *Hsp27* are highly expressed in ovaries (Figure 2B). We also noticed that high to extremely high transcription level in the central nervous system (CNS) could be assigned to all sHsps, except *CG7409* and *CG13133* (Figure 2B). In a similar way, global temporal analyses of sHsp gene expression (Figure 2A) reveal that *Hsp26*, *Hsp67Ba*, *Hsp23*, and *Hsp27* are highly expressed in early stage embryos (4–6 h after egg laying, AEL), whereas *Hsp67Bc*, *Hsp23*, *CG7409*, and *CG14207* are the only sHsps showing high expression levels during late embryogenesis (from 18 to 20 h AEL). These large scale transcriptomic datasets are consistent with previous analyses of stress-independent transcriptional regulation of sHsps [12]. It has been found that sHsp genes are, in general, kept in an active chromatin environment to allow efficient heat shock factor (HSF)-dependent but also HSF-independent transcription. All *Drosophila* Hsp promoters contain GA dinucleotide repeats that bind GAGA factors known to promote open chromatin configuration [13], thus facilitating transcriptional activation of sHsp genes. In the case of *Hsp26* promoter, GAGA factors are also involved in RNA polymerase pausing [14] in non-stress conditions, and play a role in quick transcriptional induction. *Hsp26* also presents an interesting model of DNA loop formation, where the DNA segment separating the two HSF-binding elements (HSEs) is wrapped around a nucleosome, allowing cooperative interactions between HSFs [15]. Regarding transcriptional regulation during development, it has been found that ovarian induction and larval/prepupal sHsp expression is regulated by ecdysone via elements distinct from HSEs [16,17,18,19,20,21,22,23,24].

**Figure 3:**
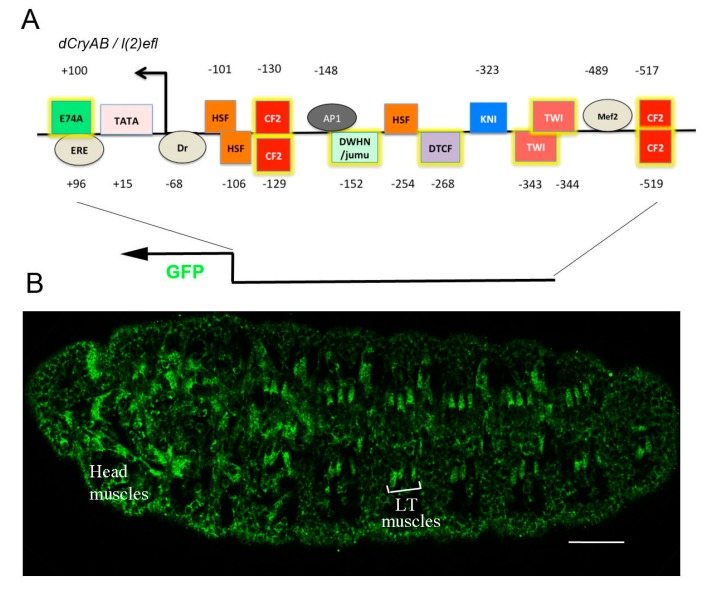
Example of muscle-specific expression of dCryAB. (**A**) dCryAB regulatory region contains several binding sites for muscle/mesoderm transcription factors, including TWI, CF2, and Mef2. Others indicated in the scheme binding sites correspond to E74A—Ecdysone-induced protein 74; ERE—Ecdysone response element; Dr—Drop/Msh; HSF—heat shock factor; AP1—Jra transcription factor; DWHN/Jumu—Jumeau; DTCF—Wingless effector; KNI—Knirps. (**B**) Presented in (**A**) 5′ dCryAB regulatory region cloned upstream of GFP reporter gene drives expression in somatic muscles. Presented is a lateral view of late stage dCryAB–muscleEnh–GFP embryo. GFP expression is detected in lateral transverse (LT) and a subset of head muscles. A single optical section is shown. Scale bar 40 µm.

In vertebrates, transcriptional regulation of several sHsps has been analyzed, allowing identification of tissue-specific developmental enhancers. For example, showing high expression in lenticular tissues, αB-crystallin (CryAB) carries two lens-specific regulatory regions (LSRs) [25] with binding sites for Maf, Pax, and RAR/RXR transcriptional regulators known to regulate eye development. CryAB also displays expression in non-lenticular tissues, including developing muscles potentially regulated by E-box carrying a conserved enhancer located immediately upstream of the promoter sequence [12]. Myogenic regulatory factors (MRFs), such as MyoD, could bind to the MRF element and drive CryAB expression in muscle [26]. Importantly, muscle-specific expression of dCryAB is evolutionarily conserved [7], and our unpublished data show (Figure 3A) that the sequences located between positions −520 and +100 of dCryAB contain several consensus binding sites of myogenic factors, including TWI, CF2, and Mef2, and drive reporter gene expression in transgenic embryos, specifically in developing muscles (Figure 3B).

Thus, stress-independent regulation of sHsp gene expression in *Drosophila* appears to be under the control of key developmental regulators. Below, we discuss some examples of developmentally regulated expression and functions of *Drosophila* sHsp genes, with a particular focus on sHsps that are activated in gonads, the developing nervous system, and muscles.

## 4. sHsps in the *Drosophila* Reproductive System

**Figure 4:**
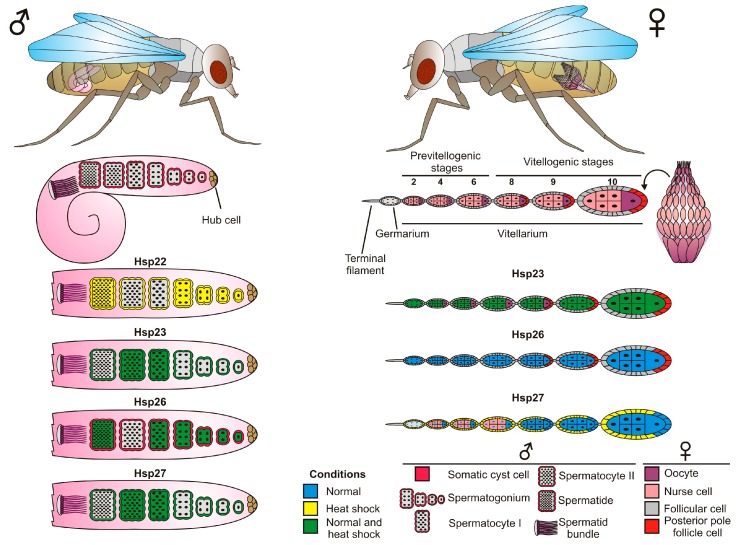
Developmentally regulated and heat shock-induced expression of Hsp22, Hsp23, Hsp26, and Hsp27 in male and female *Drosophila* germ line cells.

*Drosophila*, like other terrestrial insects, is highly exposed to changes in environmental conditions. In order to achieve reproductive success following environmental stresses (e.g., thermal changes) animals develop many molecular mechanisms to protect the germline cells exposed to stress. As shown in Figure 2B and Figure 4, the expression of several sHsps is tightly associated with germline cell development, strongly suggesting protective roles.

### 4.1. Ovary

The female reproductive system of *Drosophila* consists of a pair of ovaries, which are composed of 12–16 ovarioles. The two ovaries are connected to lateral oviducts; these merge in a common oviduct that leads to a vagina. Each ovariole is composed of four elements: terminal filament, germarium, vitellarium, and the pedicel. The terminal filament is the anteriormost part of the ovariole, composed of stacks of somatic cells. In the germarium, germline stem cells undergo asymmetric mitoses to give rise to cystoblasts of the ovarian follicles (egg chambers). Subsequently, the cystoblasts, after four mitotic divisions with incomplete cytokinesis, form an interconnected 16-cell germline cyst. In the vitellarium, each germline cyst is surrounded by somatic follicle cells. In an ovarian follicle, one of the cyst cells differentiates into an oocyte, and the remaining 15 cells become nurse cells or trophocytes. The last part of the ovariole, the pedicel, is composed of somatic cells, and joins the ovariole to the lateral oviduct [27,28] (Figure 4).

The diagram shows the expression patterns of selected sHsps in male and female *Drosophila* germline cells. Simplified diagrams of *Drosophila* gonads (testicles and ovaries) are presented, so that the nuclei of the follicular cells, as well as the perinuclear region of the nurse cells, are not marked. Different types of male and female germline cells are indicated by different shapes (male) and colors (female). Somatic cells of the reproductive system—cyst cells in male and follicular cells in female—are also shown. The “conditions” refers to the expression pattern of specific sHsps. “Normal” (blue) corresponds to constitutive expression pattern, “heat shock” (yellow) states for expression induced by increased temperature, whereas “normal and heat shock” (green) concerns a situation in which a particular sHsps are expressed in both cases.

Among sHsps, expressions of *Hsp23*, *Hsp26*, and *Hsp27* were found to be developmentally regulated during oogenesis. *Hsp27* was observed in nurse cells and oocytes [29], and was localized in the nuclei of nurse cells in the germarium up to stage 6, and was then perinuclear and cytoplasmic from stage 8. *Hsp27* was also expressed in the nuclei of the posterior pole follicle cells at stage 8–10. After a heat shock, *Hsp27* was found mostly in somatic follicle cells surrounding the germline cysts both in the germarium and vitellarium. It has been postulated that *Hsp27* could control cell division and/or differentiation of germ cells during normal growth, and help maintain ovarian integrity under environmental stresses [29]. These roles may be modulated by different mechanisms, including regulation of the stage-dependent intracellular localization and by *Hsp27* phosphorylation [29]. Like in *Drosophila*, intracellular localization of the *Hsp27* also changes during oogenesis of the Mediterranean fruit fly (*Ceratitis capitata*) [30]. In the early stages, it is located in the perinuclear region of the nurse cells and in the cytoplasm of the follicle cells and, in the late stages, is associated only with the nuclei of somatic cells. Nuclear localization of *Hsp27* in cells with elevated transcription suggests it could be involved in RNA synthesis and/or processing.

*Hsp23* and *Hsp26* exhibit distinct spatial and temporal patterns of expression [24,31]. *Hsp26* mRNA is present in nurse cells and later in the oocyte, and recent studies [32] reveal that maternal loading of the *Hsp23* plays an important role in thermal tolerance: overexpression of *Hsp23* in female oocytes significantly increased thermal tolerance in offspring embryos and larval performance [32]. Marin et al. [33] reported that, in head and testes, *Drosophila Hsp23* is constitutively expressed in two forms (native *Hsp23a* and more acidic *Hsp23b*). Only form “a” of *Hsp23* is present in unstressed ovaries, whereas *Hsp23b* isoform is induced when ovaries are submitted to a heat shock. According to Marin et al. [33], the presence of distinct isoforms of sHsps indicates that each individual sHsp may have distinct mechanisms of posttranslational modifications, and that this modulation may be important for cell-specific function.

Altogether, besides thermoprotective functions, developmentally regulated expression of sHsps during oogenesis indicates that they help safeguard both proliferative and differentiated germline states.

### 4.2. Testes

The adult testes are the main component of the *Drosophila* male reproductive system. They form a pair of coiled tubes in the abdominal part of the body cavity (Figure 4). The testes develop in late embryogenesis, and are formed by germ and somatic gonadal cells. The main function of this organ is to produce sperm. This occurs through stem cells located at the apical tip of the testis, which divide and differentiate into spermatids in a process termed spermatogenesis [34,35].

The *Drosophila* sHsps are synthesized in specific cells of the germline, and in some somatic parts of the male reproductive system [31,36]. Their expression in the male reproductive system has been detected at different developmental time points, including larval, pupal, and adult stages [24,31,36,37].

*Hsp26* is expressed in germline cell types including primary spermatocytes, and in some spermatogonia and spermatids [31,38]. Its spermatocyte-specific expression is mediated by unique regulatory elements [17,38].

*Hsp23* expression can be detected predominantly in somatic cells, such as cyst cells, and the epithelial cells of the testis and of the seminal vesicle. *Hsp27* is expressed not only in gonadal somatic cells and epithelial cells of the accessory glands, but also in the maturing spermatocytes of the germline. Worth noting, stress conditions (heat shock) did not change the location of the expression of these proteins. Contrary to this, the induction of heat shock in male gonads caused that the cells forming them to acquire the ability to express other small heat shock proteins, such as *Hsp22*. This protein is not expressed during development in the testes [20]. For its expression, an HSF is necessary, the availability of which is restricted to specific cells, such as cyst cells, epithelial pigment cells, spermatogonia, and spermatids, but not the primary spermatocytes. The heat shock has no significant effects on the levels of *Hsp23*, *Hsp26*, and *Hsp27* expression in testes [20].

Although the function of the *Drosophila* sHsps in spermatogenesis remains to be elucidated, the differences in their spatiotemporal pattern of expression suggest they may exercise cell-specific functions. Data from other species offer some clues: for example, in mammals, *Hsp27* has been shown to be involved in regulation of the meiotic prophase in the testicular germ cell [39] and, in humans, its expression in testes also indicates that it may be associated with male germ cell development [40].

## 5. Functions of sHsps Expressed in the Developing Nervous System

In *Drosophila* embryos, the CNS develops from ventral neurogenic ectoderm. This process begins by the delamination of neuroblasts (NBs) (30 per hemisegment), that divide asymmetrically and are responsible for the diversity of neuronal and glial cell lineages, each of which can be identified by specific position, morphology, and the molecular markers they express. Some neural lineages, such as MP2 lineage, play scaffolding roles and allow proper connectivity between the neuronal networks. More generally, the function of the CNS is to collect and interpret environmental information and control animal behavior. Compared with the adult fly, *Drosophila* larvae have a relatively simple information-collecting sensory system, mainly dedicated to mechanoreception and to controlling locomotor behavior during crawling. This is achieved via neuromuscular connections termed neuromuscular junctions (NMJs) that are key structures at the interface of neural and muscular systems. Interestingly, as discussed below, CNS-specific developmental expression of sHsps has been detected in structurally important MP2 lineage, and is crucial for animal mobility NMJs.

Among sHsps with high transcript levels in the developing CNS (Figure 2B), embryonic expression and function of *Hsp23* has been precisely analyzed [21]. *Hsp23* was found to be expressed in a stage-specific manner by a restricted subset of cells of the embryonic CNS. In early-stage embryos, in addition to the cephalic neuronal cell population, *Hsp23* is activated in Ftz-positive midline neuroblasts of MP2 lineage, and a single lateral chordotonal organ precursor [21]. Later during embryonic development, Hsp23 could be detected in two MP2 daughter cells but, also, in a novel group of ventral neural cells, the ventral unpaired median (VUMs) neurons and the posterior midline glia (MGP). Finally, in late-stage embryos, as determined by co-localization with Sli-lacZ marker, Hsp23 expression becomes restricted to midline glial cells [21]. This restricted expression pattern suggested a potential role of Hsp23 in specification and/or differentiation of MP2, VUMs, and MGP lineages, which play important roles in setting the commissural architecture in the ventral cord of the CNS. However, no obvious defects in transversal commissure separations and in longitudinal commissural tracts were detected in embryos homozygous for null *Hsp23* mutation, suggesting that MP2, VUMs, and midline glia form normally in the absence of *Hsp23*. Also, ubiquitous (Actin–Gal4) and neural (Elav–Gal4) overexpression of *Hsp23* has no effect on central and peripheral nervous system development [21]. Altogether, these data are evidence that Hsp23 function is dispensable for neurogenesis. However, the fact that Hsp23 is expressed in pioneer neurons of longitudinal tracts (MP2 daughters) and in VUMs, ensuring proper separation of transversal commissural tracts, suggests that it could play a beneficial role for the organism by making these neural cells with vital functions more resistant to environmental insults [21].

Like Hsp23, Hsp26 also falls into the sHsp group with high CNS expression (Figure 2B) and is one of the sHsps with an essential developmental role [2]; as revealed by pan-neural knockdown, Hsp26 plays a role in neural development and in particular in the proper formation of NMJs. The *Hsp26* loss of function results in an adversely affected presynaptic cytoskeleton, and a reduced number of synaptic buttons at NMJs. Consistent with global tissue and developmental time datasets (Figure 2), our recent work [8] revealed that Hsp67Bc is also expressed at the level of NMJs during larval development. The human ortholog of *Hsp67Bc*, the *HSPB8* gene, has been associated with two inherited human pathologies, autosomal dominant dHMN (K141E variant) [41] and CMT disease type 2L (K141N variant) [42]. We generated *Drosophila* models of these variants, and found that NMJs expressing K141N variant displayed an aberrant morphology [8], suggesting a functional impact of Hsp67Bc on proper NMJ development.

The fact that Hsp26 and Hsp67Bc are expressed at the level of NMJs and promote their proper formation points not only to beneficial roles as chaperones for vital structures, such as NMJs, but also to specific functions of sHsps in developmental processes.

## 6. Roles of sHsps Expressed in Developing Muscles

### 6.1. Drosophila l(2)efl/dCryAB Displays Stress-Independent Expression in Developing Larval Body Wall Muscles, and Is Required for Their Structural Integrity

*Drosophila* muscles, like neural cells, develop from a restricted population of progenitor cells that undergo asymmetric divisions to generate muscle founder cells (FCs). About 30 FCs are specified in each embryonic hemisegment, and are recognizable by their spatial positioning and expression of specific markers. Each FC fuses with a defined number of fusion-competent myoblasts (FCMs) to form multinucleated body wall muscle precursors. During larval life, body wall muscles grow to acquire their contractile functions and ensure optimal larva mobility. This process involves assembly of myofibrils, functional muscle subunits built of repeated contractile blocks called sarcomeres. Each sarcomere is composed of thin actin and thick myosin filaments, and a set of associated sarcomeric proteins. For example, actin filaments are associated with tropomyosin (Tm)/troponin (Tn) protein complexes but, also, with α-actinin, titin, and Zasp52, all anchored at the sarcomeric Z-line. Myofibrils are interconnected and stabilized by intermediate filament (IF) proteins that form Z-disc bridges. The locomotor muscle system and, in particular, its complex contractile apparatus needs to be well protected, and some sHsps play active developmental roles in this protection, acting to keep muscles healthy [43].

**Figure 5:**
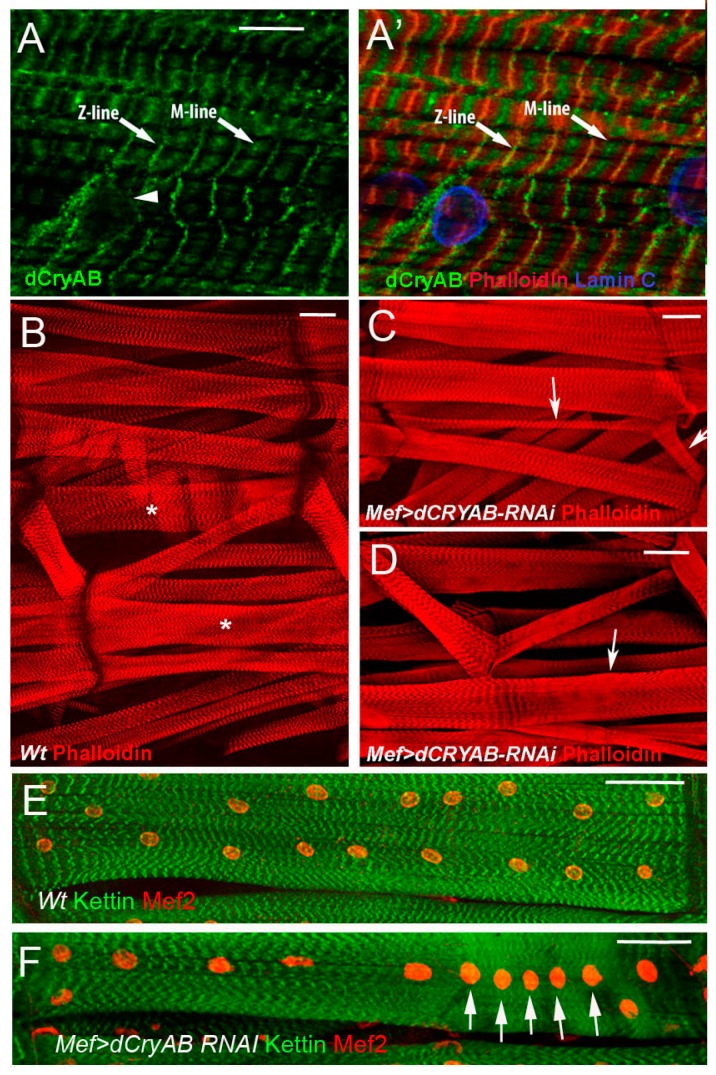
dCryAB localization in sarcomeres of larval body wall muscles and effects of muscle specific attenuation of *dCryAB*. (**A**,**A′**) dCryAB is localized in Z-line, M-line, and in the perinuclear area (arrowhead) of the muscle fiber. Scale bar, 10 µm. (**B**) Wild type muscle pattern from 3rd instar larvae revealed by the phalloidin staining. Asterisks in B mark muscles that are affected in (**C**,**D**). (**C**) Muscle splitting and (**D**) affected sarcomeric pattern in 3rd instar larvae with attenuated expression of dCryAB are indicated by arrows. (**E**) Wild type nuclei pattern in ventral VL3 muscles revealed by Mef2 antibody staining. (**F**) Nuclei clustering (arrows) observed in VL3 muscle from *Mef* > *dCryBRNAi* larvae. Scale bars, 20 µm.

Analyses performed in our laboratories [7] have identified muscle-specific expression and related functions of *l(2)efl/dCryAB* encoding the *Drosophila* ortholog of human αB-crystallin. Interestingly, in vertebrates, αB-crystallin is also expressed in muscles, where it interacts with components of intermediate filaments, such as desmin, and helps maintain cytoskeletal integrity in skeletal and cardiac muscles. Our data show [7] that, in *Drosophila*, dCryAB protein is present in all somatic muscles of 3rd instar larvae (Figure 5), and displays a striated pattern that co-localizes with F-actin in Z-discs, and with myosin in M-line. As revealed by double staining with lamin C, dCryAB protein is also observed at the periphery of myonuclei (Figure 5). This specific muscular expression prompted us to test for *dCryAB* functions. It was recently found that ubiquitous RNAi knockdown of *l(2)efl*/*dCryAB* could lead to lethality [2]. Consistent with this, muscle-specific RNAi attenuation of *dCryAB* leads to severe morphological and ultrastructural defects in body wall muscles (Figure 5). We observed affected myofibril alignment, muscle fiber splitting, and an aberrant pattern of sarcomeric actin. These defects are consistent with potential actin-binding capacities of dCryAB supported by Co-IP mass spectrometry analyses, and by the presence of a putative actin-binding domain in the dCryAB protein sequence [7]. Moreover, muscle-targeted overexpression of dCryAB without an actin-binding domain mimics *dCryAB RNAi* knockdown phenotypes (Jagla T. unpublished data), suggesting that dCryAB interacts with sarcomeric actin to stabilize myofibril organization.

Another phenotype associated with loss of *dCryAB* in muscles is the mislocalization and clustering of nuclei. Interestingly, our mass spectrometry analyses revealed that, in addition to actin, dCryAB could interact with the *Drosophila* filamin ortholog Cheerio [7].

Cheerio has already been shown to be involved in the localization of nuclei in ovarian nurse cells [44], but its role in muscles has not been tested. We found that Cheerio co-localizes with dCryAB in Z-lines and in the nuclear periphery, and that loss of *cheerio* function mimics the nuclei mislocalization and sarcomeric defects observed in *dCryAB RNAi* knockdown larvae. One hypothesis we favor is that dCryAB acts as a chaperone of Cheerio in somatic muscles, thereby stabilizing and protecting both myonuclei positioning and the contraction apparatus [7].

### 6.2. Hsp67Bc Displays Evolutionarily Conserved Developmental Muscle Expression and Function

In addition to *dCryAB*, our recent data [8] reveal that *Hsp67Bc* is also expressed in a stress-independent manner in the larval body wall muscles. Hsp67Bc protein could be detected in a striated sarcomeric pattern at the level of Z-disk and A-band. A granular pattern of Hsp67Bc expression suggests it might be part of large protein complexes. The observed *Drosophila* larvae Hsp67Bc expression pattern is reminiscent of that of its vertebrate ortholog HSPB8, [45], suggesting that it could serve analogous functions in Z-disc stability. HSPB8 is known to modulate autophagy-mediated protein degradation via the eIF2 pathway, and acts in a complex with BAG3 [46]. Interestingly, this cooperation is also present in *Drosophila* between counterparts of HSPB8 and BAG3, the Hsp67Bc and starvin proteins, facilitating the clearance of misfolded aggregate-prone proteins [46,47]. It has also been found that HSPB8 hotspot mutations, K141E and K141N, cause distal hereditary motor neuropathy (dHMN) and Charcot–Marie–Tooth disease type 2F (CMT 2F), respectively with neural and muscular involvements. Thus, to test potential muscle functions of Hspb67Bc, we generated *Drosophila* models of these hotspot mutants [8]. Ultrastructural analyses of *Drosophila* larval muscles expressing Hsp67Bc mutant proteins revealed the presence of interrupted and improperly spaced Z-disks, excessive accumulation of glycogen granules, and membrane-bound autophagosomes containing glycogen. Moreover, mitochondria were characterized by the presence of broken cristae, which suggested impaired respiratory function. As expected, this altered muscle organization affected muscle performance and reduced larva mobility [8]. These data indicate that, like dCryAB, Hsp67Bc not only protects contractile apparatus, but also plays an active role in ensuring Z-disk integrity and proper muscle function.

What seems to be particularly interesting to us is the fact that two mutations (R126E and R126N) which we introduced in the gene coding for Hsp67Bc have been associated with two slightly different phenotypes. This also occurs in humans, and suggests that the pathogenic pathway connected with the change of one residue, leading to similar but different phenotypes, might not be necessarily identical. This makes *Drosophila* a potentially useful model in the study of human pathologies with similar background.

Since our experiments involving muscle-targeted expression give us limited insight into Hsp67Bc function in other tissues, it is noteworthy to consider the performance of additional research focused on Hsp67Bc neural overexpression, which could bring valuable data. In vivo studies on the impact of the mutated forms of Hsp67Bc, involving other tissue-specific promoters in *Drosophila melanogaster* should enable finding the answers to the questions about a role of the HSPB8 ortholog in the maintenance of tissue-specific homeostasis, and mode of action of their disease-connected mutation.

However, as many studies suggest, in-depth characterization of a particular sHsp’s function may be difficult, because of their overlapping functions, as well as compensation effect.

## 7. Concluding Remarks Conclusions?

The examples of stress-independent transcriptional activity of sHsps, presented here, demonstrate that expression of these genes can be detected in organs and cells with vital functions, such as the reproductive system and the locomotor neuromuscular system. This suggests that during evolution, organisms developed specific regulatory mechanisms that switch on sHsp expression, allowing production of protective chaperones at developmental time points and in cells demanding particular safeguarding. This is the case for the finely regulated expression of Hsp23 in neural lineages that are important for building CNS architecture [21]. Here, as shown by loss of function analyses, developmentally regulated Hsp23 expression seems to have only a protective role, with no impact on CNS development. However, as suggested by developmental expression profiles (Figure 2), other sHsps are also expressed in developing embryos and in CNS, and they could potentially have redundant functions. The recent development of CRISPR-based gene knockout allowing simultaneous targeting of several genes will help resolve sHsps redundancies. On the other hand, the fact that sHsp genes are, in general, kept in an active chromatin environment, could facilitate their transcriptional activation in different developmental contexts. However, the impact of decondensed chromatin status on developmental Hsp functions remains unknown.
